# Antimicrobial Oleogel Containing Sustainably Prepared Silver-Based Nanomaterials for Topical Application

**DOI:** 10.3390/jfb15010004

**Published:** 2023-12-20

**Authors:** Valeria Ambrogi, Morena Nocchetti, Donatella Pietrella, Giulia Quaglia, Alessandro Di Michele, Loredana Latterini

**Affiliations:** 1 Dipartimento di Scienze Farmaceutiche, University of Perugia, Via del Liceo 1, 06123 Perugia, Italy; morena.nocchetti@unipg.it; 2 Nano4Light Lab, Dipartimento di Chimica, Biologia e Biotecnologie, University of Perugia, Via Elce di Sotto 8, 06123 Perugia, Italy; donatella.pietrella@unipg.it; 3 Dipartimento di Medicina e Chirurgia, University of Perugia, Piazzale Lucio Severi 1, 06129 Perugia, Italy; quagliagiulia09@gmail.com (G.Q.); loredana.latterini@unipg.it (L.L.); 4 Dipartimento di Fisica e Geologia, University of Perugia, Via Pascoli, 06123 Perugia, Italy; alessandro.dimichele@unipg.it

**Keywords:** silver nanomaterials, oleogel, antimicrobial and antibiofilm activities, cytotoxicity

## Abstract

Oleogels containing silica–silver-based nanomaterials were prepared to be used as potential antimicrobial treatment for preventing and curing skin infections. Fumed silica was used as a bifunctional excipient able to offer support to silver-based nanoparticle growth and act as a gelling agent for oleogel formulation. First, silica–silver composites were prepared following a sustainable method by contact of fumed silica and silver nitrate in the presence of ethanol and successive UV irradiation. The composites were characterized by transmission electron microscopy (TEM), scanning electron microscopy (SEM), ATR FT-IR spectroscopy and UV-Vis spectrophotometry. The presence of 8–20 nm spherical nanoparticles, in addition to the silica aggregates and AgNO_3_ crystals, was detected. The composites showed good antimicrobial activity against the Gram-negative *Pseudomonas aeruginosa* and the Gram-positive bacteria *Staphylococcus aureus* and *Staphylococcus epidermidis*. Thus, they were formulated in an oleogel, obtained using fumed silica as a gelling agent. For comparison, oleogels containing AgNO_3_ were prepared according to two different formulative techniques. The silica–silver-based oleogels showed good antimicrobial activity and did not show cytotoxic effects for fibroblasts and keratinocytes.

## 1. Introduction

The skin is one of the body’s first lines of defense against microbial invasion, but its harm can provide a pathway for Gram-positive and Gram-negative bacteria, which can cause skin and soft tissue infections [1]. Skin lesions can occur due to trauma, insects or surgical incisions, and tattooing, whose prevalence is increasing enormously, can also predispose one to skin infection problems [2].

Semisolid topical formulations, such as ointments, creams, gels and pastes, have a long-time tradition in the treatment of skin infections. In this kind of formulations, the use of classical antibiotics should be limited because of the increasing number of bacteria resistant to antimicrobial agents [3] due to inappropriate self-made therapies and to malpractice, since clinicians and patients consider topical antimicrobials less potentially dangerous than systemic drugs [4].

Therefore, some topical formulations containing silver, ZnO, TiO_2_ and CuO nanoparticles have been proposed as an alternative to formulations containing conventional antibiotics for the treatment of cutaneous infections for human and veterinary use [5,6,7,8,9,10,11]. In particular, silver-based nanoparticles may be seen as promising agents to both combat bacterial infections and prevent them. In fact, silver ions have a broad spectrum of antimicrobial and antifungal activities known from the past, and when in nanoparticle size, enhanced properties are observed [12]. On the basis of these considerations, metal-based nanoparticles represent a promising tool to fight bacterial resistance, which is one of the top three global health challenges, as affirmed by the World Health Organization [13].

Silver-based nanoparticles act through mechanisms that are completely different from those of traditional antibiotics, and show several mechanisms to exert their antimicrobial effects and target multiple biomolecules [14]. Their exact mechanism of action is, however, still under investigation, although it seems to be the result of a complex of factors, such as the following: (i) the release of free metal ions from the nanoparticle dissolution, which exert their toxic action against cell membrane and cellular biomolecules; (ii) oxidative stress via the generation of reactive oxygen species [15]; and (iii) the destruction of bacterial cell integrity by direct contact between the nanoparticle and the cell wall [16,17].

Recently, the use of microsized silver has been proposed also for the treatment of bacterial infections associated to atopic dermatitis [18]. In fact, this multifactorial skin disease is characterized by a reduction in the skin barrier function, chronic skin inflammation and higher susceptibility to bacterial colonization, especially of *Staphylococcus aureus* [19,20,21]. Treatment with moisturizing agents is an important therapeutic tool to reduce skin xerosis and maintain its hydration, and long-chain aliphatic hydrocarbons, such as petrolatum and liquid paraffin oil, are widely used [22,23]. Moreover, these excipients have been proposed to protect skin against post-ambulatory surgical local infections and are widely used for the treatment of minor surgical procedures [24].

For obtaining a topical formulation containing nanosized silver and liquid paraffin, our attention was pointed to silica for the following two reasons.

Firstly, silica acts as a support for nanoparticle preparation. In fact, colloidal and mesoporous silicas have been proposed as a support for silver and zinc oxide nanoparticles [25,26] due to the presence of free silanols on its high surface, which allows for a distribution of the metal/cation, decreasing their mobility and nanoparticles’ aggregation. Moreover, mesoporous silica can act as a template to confine the growth of silver clusters and to control nanoparticle size [27]. Finally, the presence of the support could reduce the uncontrolled dispersion of nanoparticles in the environment and in the host.

Secondly, fumed silica is a largely used multifunctional excipient which can act as a glidant for solid dosage forms, excellent absorbent for liquid active pharmaceutical ingredients, moisture scavenger for the improved storage stability of tablets, carrier for improving the dissolution of Biopharmaceutic Classification System class II drugs, and thickener for pharmaceutical oils. Fumed silica, when mixed with mineral or vegetal oils, gives rise to transparent gels with a particularly good spreading behavior [28,29].

In this paper, fumed silica has been used as a bifunctional excipient able to both obtain an oleogel and give a support to silver nanoparticles preventing their agglomeration. As a fumed silica, Cab-O-Sil H5 (afterwards called Cab-O-Sil) was used. It is characterized by extremely small particles size and large surface area (300 m^2^/g), smooth surface and interparticle porosity due to the formation of agglomerate of aggregates of silica nanoparticles [29]. The antibacterial properties of silver-based composites, obtained by a sustainable method, and their oleogel formulations were tested against Gram-positive (*Staphylococcus aureus* and *Staphylococcus epidermidis*) and Gram-negative (*Pseudomonas aeruginosa*) bacteria, and cytotoxicity was evaluated as well.

## 2. Materials and Methods

Cab-O-Sil was a gift from Cabot (Ravenna, Italy). The reverse osmosis process with a MilliQ system (Millipore, Rome, Italy) was used to obtain deionized water. Silver nitrate (AgNO_3_) and ethanol (reagent grade) were acquired from Sigma Aldrich (Milan, Italy). All excipients used for oleogel were purchased by Caelo (distributed by Comifar, Corciano, Italy).

### 2.1. Preparation of Cab-O-Sil-Ag

Silver nitrate (400 mg) was dissolved in 15 mL of ethanol and kept under magnetic stirring, and then Cab-O-Sil (1 g) was added. The reaction mixture was maintained under stirring for 24 h at room temperature. The resulting powder was recovered by filtration, dried in a stove at 37 °C for 24 h and stored in a desiccator with P_2_O_5_. The entire procedure was performed in dark conditions to prevent room and sunlight interferences.

### 2.2. Cab-O-Sil-Ag UV Irradiation

CAB-O-Sil-Ag (20 mg) was exposed to 366 nm radiation for 2 h, using a medium pressure mercury lamp provided with a band pass filter at 366 nm for selective irradiation. The obtained sample (Cab-O-Sil-Ag-Irr) was characterized, and its properties were compared to those of the non-irradiated sample to monitor the effects of light exposure on the powder.

### 2.3. Characterization of Cab-O-Sil-Ag and Cab-O-Sil-Ag-Irr

X-ray powder diffraction (XRPD) spectra were collected with a diffractometer (Bruker D8 Advance, Bruker AXS GmbH, Karlsruhe, Germany) in Bragg-Brentano geometry, equipped with a Lynxeye XE-T fast detector, CuKa radiation (operative conditions: 40 kV and 40 mA, step size 0.033° 2θ, step scan 30 s). The Bruker DIFFRAC.EVA V5 software equipped with the COD database was used for the phase identification. A Philips 208 transmission electron microscope, operating at an accelerating voltage of 100 kV, was used to carry out TEM analysis. An ethanolic dispersion of weighted powders was prepared and sonicated for a few min., then the dispersion was supported on copper grids (200 mesh) precoated with Formvar carbon films and quickly dried. FE-SEM was used for examining the morphology of the films through a LEO 1525 ZEISS instrument (Oberkochen, Germany) using an Inlens detector and AsB (Angle selective Bescattered) detector. Bruker Quantax EDX was used for determining elemental mapping. The Ag content in the composites was determined by an ICP Varian Liberty inductively coupled with a plasma-optical emission spectrometer (ICP-OES) with axial injection. A known amount of the sample (ca. 20 mg) was dissolved in 2 mL 3M HF solution for silica dissolution, then concentrated HNO_3_ (≈1 mL) was added for Ag dissolution. Finally, deionized water was added up to 50 mL. The reflectance spectra of the sample were recorded by a Varian (Cary 4000) spectrophotometer, equipped with a 150 mm integration sphere (DRA-900). A barium sulphate tablet was used as a reference. The recorded spectra were converted to absorbance spectra through the Kubelka–Munk equation. ATR-FT-IR spectra in the 4000–300 cm^−1^ frequency range were acquired with ALPHA compact FT-IR BRUKER, provided with a diamond crystal. Each spectrum, registered in the 4000–300 cm^−1^ frequency range, was the average of 30 scans with a 2 cm^−1^ resolution.

### 2.4. Preparation of the Oleogels

Oleogel-1, oleogel-4 and oleogel-5 were prepared in a mortar by stirring Cab-O-Sil or a mixture of Cab-O-Sil and the proper silver composites (Cab-O-Sil-Ag or Cab-O-Sil-Ag-Irr) with liquid paraffin to obtain a homogenous transparent mixture. Then, the oleogel was mixed with a conditioning mixer (Thinky ARE-250, Thinky Corporation, Tokyo, Japan) at room temperature for 15 min.

For the preparation of oleogel-2, AgNO_3_ and Cab-O-Sil were properly mixed in a mortar to obtain a homogeneous mixture, then liquid paraffin was added under stirring and finally the oleogel was mixed, as previously described.

**Scheme 1:**
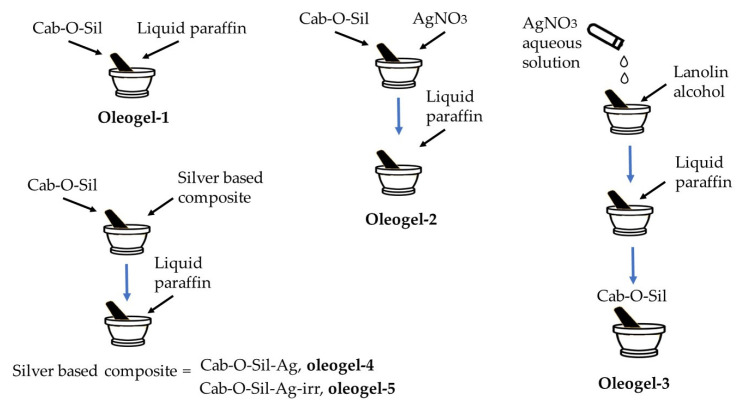
Schematic representation of oleogel preparation.

For the preparation of oleogel-3, AgNO_3_ was dissolved in the smallest amount of deionized water, the solution was incorporated into previously fused lanolin alcohols and then the mixture was mixed with liquid paraffin. Finally, the oleogel was obtained by adding this mixture, under stirring, to Cab-O-Sil and by mixing all as previously described. The composition of all oleogels is reported in Table 1, and a schematic representation of their preparation can be seen in Scheme 1.

### 2.5. In Vitro Silver Release Studies

In vitro silver release from all formulations was carried out for 24 h by a 20 mm diameter Franz diffusion cell (PermeGear, Inc., Bethlehem, PA, USA). This cell is composed of a water-jacketed receptor chamber (15 mL) and a donator chamber. The receptor phase was deionized water at 32 °C and stirred at 600 rpm. A cellulose filter membrane (Filter paper Whatman 41, 20–25 µm; Whatman GmbH, Dassel, Germany) was placed between the two chambers. Each oleogel (1 g) was placed on the upper donor chamber which was then sealed with Parafilm^®^ (Carlo Erba Reagenti S.p.A., Arese, Milano, Italy). Samples of 4 mL of the receiving phase were withdrawn at prefixed time intervals and the same volume of deionized water was added. The withdrawals were diluted with conc. HNO_3_ (1 mL) and, when required, were further diluted with deionized water. Finally, the silver content was determined by ICP. The experiment was performed three times and the data are reported as an average ± standard deviation (SD).

### 2.6. Microbial Strains and Growth Conditions

Two Gram-positive bacteria *Staphylococcus aureus* (ATCC 29213) and *Staphylococcus epidermidis* (ATCC 12228) and one Gram-negative bacteria *Pseudomonas aeruginosa* (ATCC 15692) were used as microbial strains. The bacterial cultures were maintained in Muller Hinton Agar (MHA), and one colony was inoculated in Muller Hinton broth (MHB) and incubated for 24 h at 37 °C, just the day before the test. Microbial cells were collected by centrifugation, and then washed and counted by spectrophotometric analysis. Finally, the cells were resuspended to the desired concentration in the appropriate culture medium.

### 2.7. Antimicrobial Activity of Nanocomposites

The minimum inhibitory concentration (MIC) of the composites against bacterial strains was evaluated following the Clinical and Laboratory Standards Institute (CLSI) method by broth microdilution assay using two-fold serial dilutions in MHB and 96-well U-bottom microdilution plates. Microbial inocula were obtained by subculturing bacteria into MHB for 18 h and then diluting up to ca. 105–106 CFU/mL in MHB. The test compounds (100 µL, initial concentration of 10 mg/L) were serially diluted 1:2 in an appropriate culture medium and placed in a 96-well tissue culture plate. One hundred µL aliquots of the tested bacteria were added to each well. Finally, the microplates were incubated at 37 °C overnight. The wells that were inoculated with microorganisms but free of the tested compound were used as the positive growth control, whereas gentamicin was used as the positive control. MIC is defined as the lowest concentration of an antimicrobial that inhibits the visible growth of a microorganism after overnight incubation. Each experiment was repeated at least three times.

### 2.8. Antimicrobial Activity of Oleogels

Oleogel-4 and oleogel-5 were tested for their antimicrobial activity using a disk-diffusion test (Kirby–Bauer Clinical Laboratory Standards Institute classification) in MHA, and their activity was compared to those of oleogel-1, -2 and -3. The zone of inhibition (ZOI) was measured following the previously described method with some suitable modifications [25,30].

The bacterial cells were seeded on MHB medium and left overnight at 37 °C. The bacteria were centrifuged at 2000× *g* in sterile saline buffer (PBS). Then, the bacteria were added to 2 mL of PBS and the cell concentration was determined by spectrophotometric measurement (600 nm). A bacterial suspension of 1 × 10^8^ CFU/mL in sterile phosphate buffer was uniformly spread on a Muller Hinton Agar (MHA) plate. The oleogels (100 µL) were placed into 6 mm diameter wells previously made in the agar plate. The plates were then incubated at 37 °C overnight. Gentamicin disk (20 µg) was used as a positive control. The ZOI was determined by measuring the diameter of the clearing zone around the oleogels using a metric ruler. All experiments were repeated three times (*n* = 3) and the data were expressed as mean ± standard deviation (SD).

### 2.9. Oleogel Cytotoxicity

The cytotoxicity of oleogel was tested on human fibroblasts and keratinocytes by the determination of the cell ATP level ViaLight^®^ Plus Kit (Lonza, Milan, Italy). The ATP that is present in all metabolically active cells and this method is based upon the bioluminescent ATP measurement using the enzyme luciferase which catalyzes the formation of light from ATP and luciferin. The emitted light intensity is linearly related to the ATP concentration and is measured using a luminometer. Human dermis fibroblast (HuDe) and human skin keratinocytes (NCTC2544) cells were used. These cell lines belong to the cell bank of the Department of Medicine and Surgery of the University of Perugia, Italy. HuDe and NCTC2544 were grown to confluence in RPMI 1640 supplemented with 10% heat-inactivated fetal calf serum, 10,000 units of penicillin and 10 µg of streptomycin/mL overnight. Monolayer cells were then treated for 4 and 24 h at 37 °C with 1:2 scalar concentrations of tested compounds starting from 1000 µg/mL. After incubation, the plates were allowed to cool at room temperature for 10 min and then ATP was extracted from live cells by adding the Cell Lysis Reagent to each well. Next, the AMR Plus (ATP Monitoring Reagent Plus) was added and the luminescence was read after 2 min by a microplate luminometer (TECAN). The results were expressed as cytotoxic concentration (CC_50_), which is defined as the concentration required to reduce the live cell number by 50%, compared to the untreated controls.

## 3. Results and Discussion

### 3.1. Composite Preparation and Characterization

The literature reports that silica materials are valuable matrices to support and assist the preparation of silver-based composites [25,30], and the presence of chemical defects can act as a weak reducing agent enable to reduce (at least partially) the Ag^+^ adsorbed on the silica surface. In the present work, after the adsorption of Ag^+^ on the silica support (Cab-O-Sil) from ethanolic AgNO_3_ solutions, the obtained sample was exposed to 366 nm radiation to activate the silica defects and assist the formation of silver nanoparticles.

**Figure 1:**
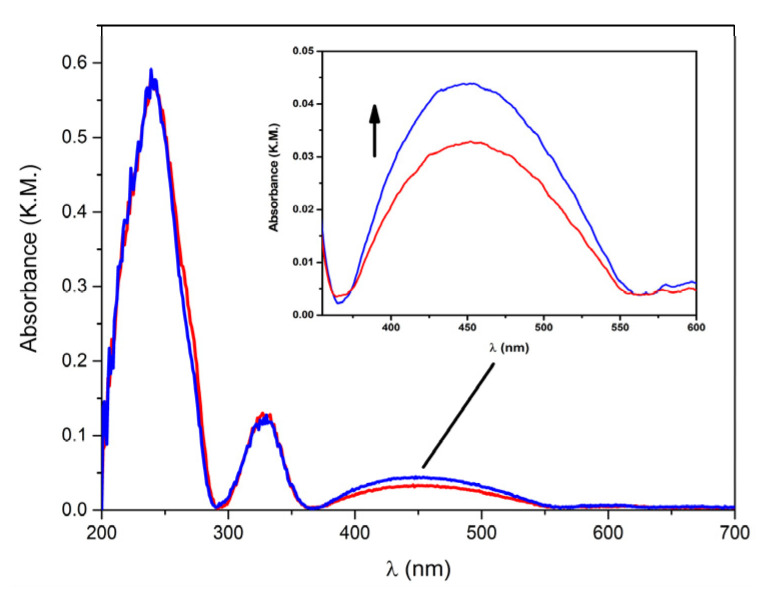
UV-Vis absorbance spectra of Cab-O-Sil–Ag (red line) and Cab-O-Sil–Ag-irr (blue line).

The optical properties of Cab-O-Sil materials with Ag^+^ adsorbed (Cab-O-Sil-Ag) and then irradiated (Cab-O-Sil-Ag-irr) have been investigated through UV-vis spectrophotometric measurements in order to verify the presence of Ag nanostructures. The reflectance spectrum of Cab-O-Sil-Ag-irr is reported in Figure 1 and compared to that of Cab-O-Sil-Ag before UV irradiation. Cab-O-Sil, used as a support, has a maximum absorption in the UV region up to 320 nm and does not show significant contributions in the visible region (see Supplementary Material Figure S1). The absorption spectrum of Cab-O-Sil-Ag shows a notable absorption band in the visible range, centered at 460 nm, which is attributable to the surface plasmonic resonance (SPR) of silver nanoparticles. After irradiation at 366 nm, the intensity of the SPR band increases, demonstrating the occurrence of the photoactivation process [30].

Information on the silver crystalline phases formed in the composites have been obtained by the XRD collected before and after UV irradiation (Figure S2). The XRD patterns have been compared with that of a physical mixture obtained by grinding in a mortar the Cab-O-Sil and AgNO_3_ in the same weight ratio of the composites. In all samples, only the orthorhombic phase of AgNO_3_ (COD 1509468) is present, suggesting that a part of silver crystallizes on the Cab-O-Sil surface as silver nitrate. Silver nanoparticles are not detected, which is very likely due to their very small dimensions or the lack of crystallinity.

**Figure 2:**
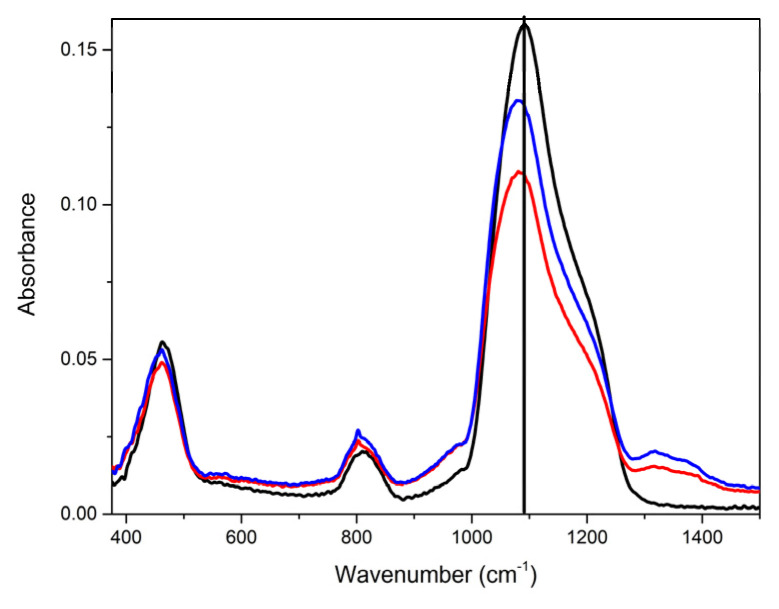
ATR-IR spectra of Cab-O-Sil (black line), Cab-O-Sil-Ag (red line) and Cab-O-Sil-Ag-irr (blue line) in the spectral range: 1500 cm^−1^–300 cm^−1^.

The ATR-IR analysis (Figure S3) has been performed to characterize the nanocomposite and the results in the spectral range of 1500 cm^−1^–300 cm^−1^, as shown in Figure 2. Cab-O-Sil presents an intense peak at 1091 cm^−1^, which is associated to the stretching Si-O-Si, and at 812 cm^−1^ the bending vibration of Si-O-Si can be appreciated. The deviation at a lower wavenumber, marked by a line, suggests that the interaction between Ag and the matrix and the shift is more evident after UV irradiation. At 1350 cm^−1^, the band observable after the adsorption of AgNO_3_ is due to the interaction between NO_3_^−^ and Si-O-/Si-OH [31].

**Figure 3:**
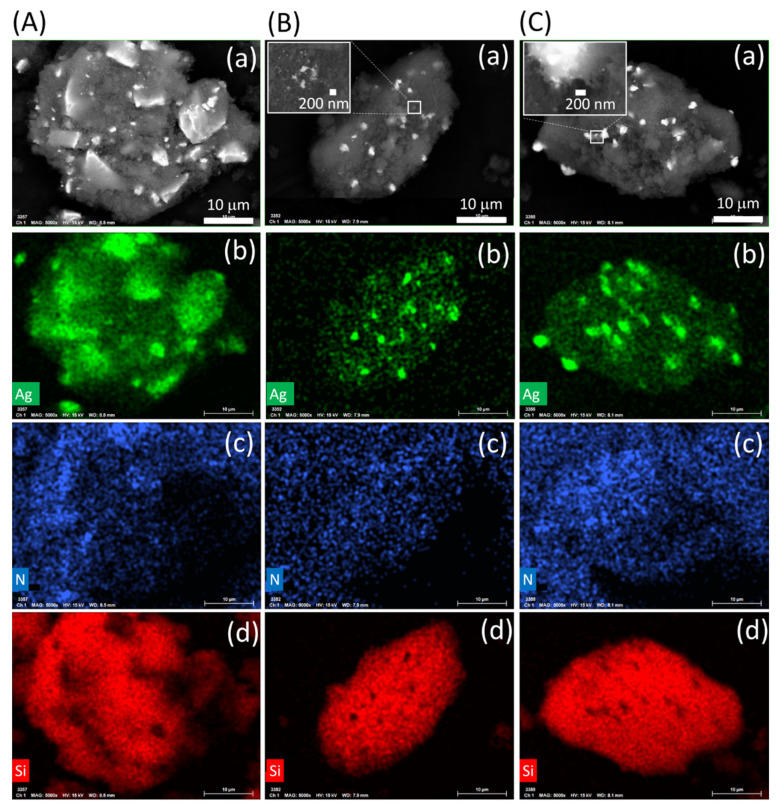
SEM recorded with angle selective backscattered (AsB) detector (**a**) and EDX images of Ag (**b**), N (**c**) and Si (**d**) of Cab-O-Sil/AgNO_3_ physical mixture (**A**), Cab-O-Sil–Ag (**B**) and Cab-O-Sil–Ag-irr (**C**).

The SEM image of the Cab-O-Sil/AgNO_3_ physical mixture (Figure 3A) shows the presence of big crystals, with a dimension ranging from 10 to 1 µm in the silica matrix; the nature of the crystals is derived from elemental mapping: the areas occupied by the crystals are rich in silver and nitrogen, allowing us to assign these crystals to AgNO_3_ according to the XRD. The composites Cab-O-Sil–Ag (Figure 3B) and Cab-O-Sil–Ag-irr (Figure 3C) are covered by crystals of AgNO_3_ of about 2 µm, accompanied by the presence of silver particles with nanometric dimensions (see inset of Figure 3B,C).

**Figure 4:**
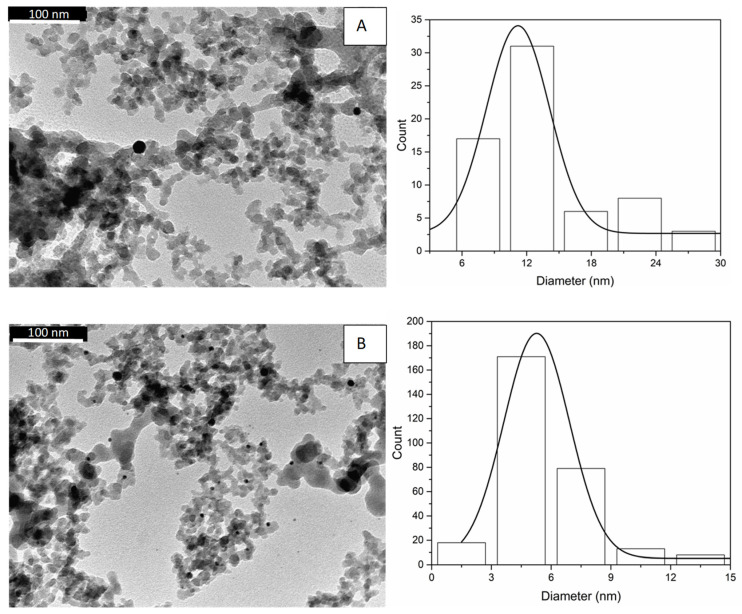
TEM images and size distribution of Cab-O-Sil-Ag (**A**) and Cab-O-Sil-Ag-irr (**B**).

A further investigation on the silver nanoparticles has been performed by TEM. A TEM image of Cab-O-Sil (Figure S4) shows its typical three-dimensional branched chain aggregates. In the Cab-O-Sil-Ag image (Figure 4), 8–20 nm spherical nanoparticles can be observed in addition to the silica. The images clearly reveal that nanoparticles form and grow on the silica surface. After UV irradiation, the image displays an overall decrease in the sizes of silver nanoparticles. The effect may be due to the fragmentation of the previously formed nanoparticles or to the further occurrence of the photoreduction process assisted by the radiation.

The silver content in the composites, measured by ICP, resulted 8.5% and 8.4% for Cab-O-Sil-Ag and Cab-O-Sil-Ag-irr, respectively.

### 3.2. Oleogel Formulation and Characterization

When Cab-O-Sil is dispersed in a non-aqueous liquid, gelation occurs due to the formation of a three-dimensional interparticle network. In fact, silanol groups which are present on the colloidal silicon dioxide form hydrogen bonds with each other with the formation of a temporary three-dimensional network, which is macroscopically visible as an increase in the viscosity of the gel compared to the pure liquid. Among lipophilic liquids, such as vegetable oils and Vaseline oil, the last one has been chosen due to its great chemical stability also in the presence of metals, which are inductors of the peroxidation reaction typical of vegetable oils. Five different oleogels were prepared, as described in Table 1. The silver concentration was 0.01%. Soon after the preparation, all oleogels were transparent and colorless, except for oleogel-3, which was opaque and yellowish (Figure S5) due to the silver oxidation in the presence of water. After one month of storage at room temperature, oleogel-3 showed the presence of syneresis, whereas in other oleogels, no color/transparency changes nor syneresis have been observed (Figure S6).

**Figure 5:**
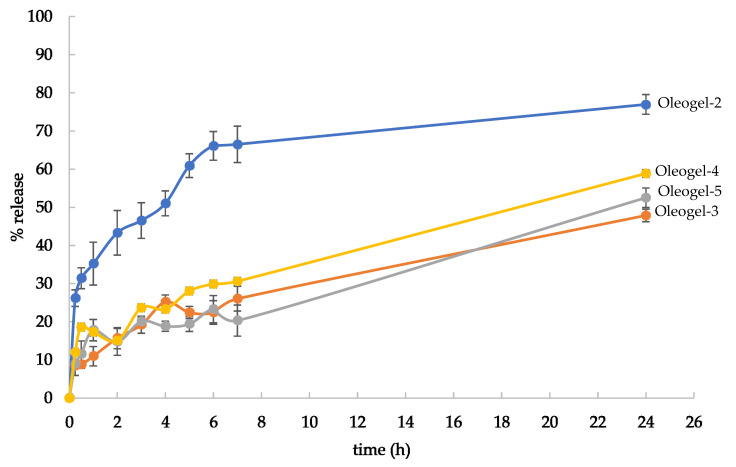
In vitro silver release from oleogels.

The silver ions’ release from prepared oleogels has been evaluated in water and the release profile is reported in Figure 5. The silver release is gradual and slow for all oleogels, but some differences are observed.

**Scheme 2:**
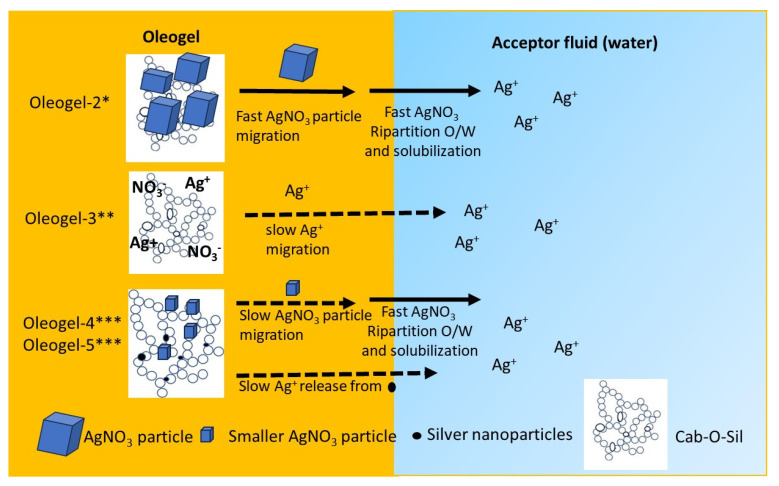
Schematic representation of silver release from the oleogels. * AgNO_3_ crystals dispersed in the oleogel; ** AgNO_3_ aqueous solution adsorbed in lanoline alcohol and incorporated in the oleogel; *** Smaller AgNO_3_ crystals and silver nanoparticles supported on silica dispersed in the oleogel.

The amount of silver released from oleogel-2 is higher than that from other oleogels, and the acceptor fluid shows that the silver ions’ concentration was always higher than those obtained from the other oleogels during the entire test. This can be due to the good solubility of AgNO_3_ in water. In fact, this salt is dispersed in the oleogel-2; thus, its particles migrate towards the interface oleogel/acceptor fluid and then rapidly dissolve in water. At the end of the test, the silver release from oleogel-2 reached ca. 77%. In the case of oleogel-3, AgNO_3_ is solubilized in water and then incorporated in the lipidic phase, and thus, this salt has to move from the aqueous phase to reach the water/oil interface and share out between the water/oil phases and then diffuse through the oily phase and reach the acceptor fluid. Because of its good water solubility and very low solubility in the lipidic phase, it has a low tendency to leave the internal aqueous phase. This can explain the slower silver release from oleogel-3 in comparison to the silver release from oleogel-2. As concerns oleogel-4 and oleogel-5, the silver release profiles were quite similar; after 24 h, a plateau was not reached and only 59% and 52% of the silver amount had been released. This slow silver release can be due to the presence of the silica-supported silver. In fact, silver nitrate migration towards the oleogel surface can be slowed down by the interaction between Ag and the matrix, as shown by ATR FT-IR, and also by their smaller size in comparison to the physical mixture, as shown in Figure 3. Moreover, the oxidative dissolution of the silver ions from nanoparticles occurred when silver is in the form of metallic nanoparticles [32,33], whose presence is proved by UV-Vis spectrophotometry. A schematic representation of silver ions’ release from the oleogels is reported in Scheme 2.

### 3.3. Antimicrobial Activity and Cytotoxicity

The antibacterial activity of the composites was evaluated by determining the MIC values against *S. epidermidis*, *S. aureus* and *P. aeruginosa*, which are typical skin-colonizing bacteria responsible for skin infections [1]. Silica was tested as well, and the results are reported in Table 2. The tested composites are not composed by silver nanomaterials but are silver-based materials and silica (Table 1); thus, MIC values were expressed for each composite in two different ways: MIC values for the composite (MIC) and MIC values normalized, which referred to the effective silver content (MIC_silver_). For determining the MIC_silver_, the following formula was used:

MIC_silver_ = (MIC × silver %)/100

Whereas silica support did not show any inhibitory activity, the prepared composites resulted active against all tested bacteria. The MIC_silver_ of the composites were slightly lower than the MIC_silver_ of AgNO_3_ for all Gram-positive bacteria (three times lower against *Staphylococcus epidermidis*, almost twice lower against *Staphylococcus aureus*). In the case of the Gram-negative *Pseudomonas aeruginosa*, the MIC_silver_ values of the composites were slightly higher than AgNO_3_ MIC_silver_.

The cytotoxicity of composites was analyzed by testing the viability of human dermal fibroblasts and human keratinocytes treated with samples for 4 and 24 h. The results, expressed as the nanocomposite concentrations normalized for silver content able to induce the CC_50_, are reported in Table 3 and are compared to those obtained for AgNO_3_.

All composites resulted less cytotoxic than AgNO_3_, both after 4 and 24 h and against both the cell lines, and CC_50_ resulted higher than MIC silver values. Very small differences could be detected between Cab-O-Sil-Ag and Cab-O-Sil-Ag-irr. It deserves to be highlighted that CC_50_ resulted higher than MIC silver values.

**Figure 6:**
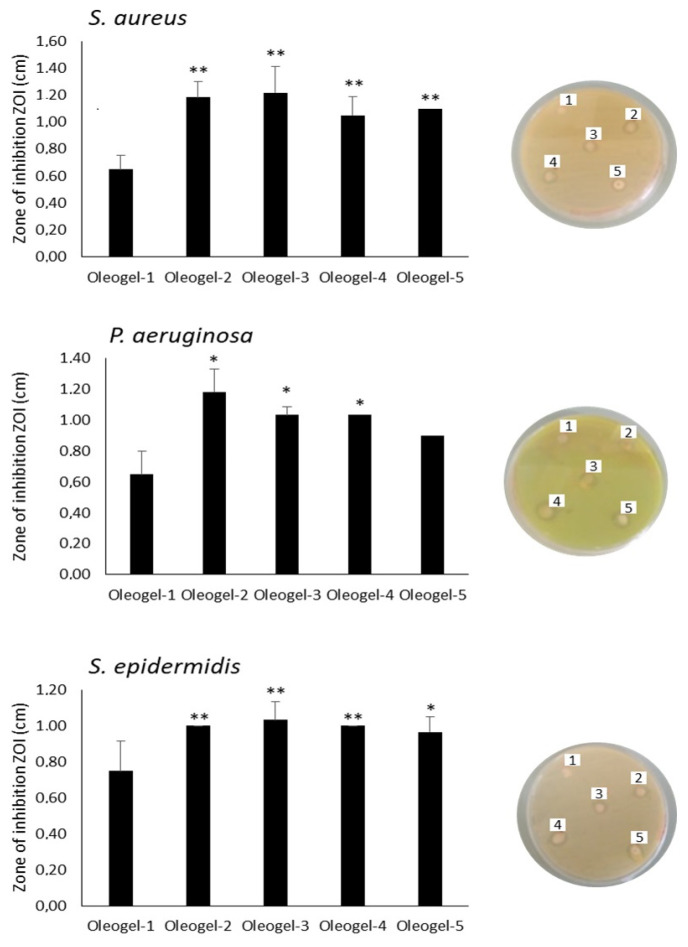
Results are reported as the average ± SD of values obtained from three independent experiments. The statistical significance was evaluated with the Student’s *t*-test (two-tailed). * *p*-values of <0.05, ** *p*-values of <0.01 were considered significant. The results reported in the picture are representative of one of the three independent experiments (1 = oleogel-1, 2 = oleogel-2, 3 = oleogel-3, 4 = oleogel-4 and 5 = oleogel-5). The cytotoxicity of the oleogels has been analyzed by testing the viability of NCTC2544 and HuDe cell lines after 4 h and 24 h, and CC_50_ values are reported in Table 4. The oleogels resulted no cytotoxic until the highest tested concentrations (250 µg/mL) after 24 h of contact. When the time contact was 4 h, oleogel-2 resulted the most cytotoxic oleogel with a CC_50_ of 136.0 µg/mL and 75.2 µg/mL for keratinocytes and fibroblasts, respectively, probably due to its higher silver release in comparison to other oleogels, as shown in Figure 5.

The antibacterial properties of oleogels 2-5 were determined using a modified Kirby–Bauer test and compared to those of oleogel-1 (Figure 6). The oleogel-1 did not show antimicrobial activity against the Gram-positive *Staphylococcus aureus* and *Staphylococcus epidermidis* and the Gram-negative *Pseudomonas aeruginosa*. The oleogels containing silver nanoparticles showed antimicrobial activity against all tested microorganisms. Oleogel-5 was active only against Gram-positive bacteria.

## 4. Conclusions

In this study, fumed silica has been used as a double functional excipient. In fact, it was used as a gelling agent for liquid paraffine and as a support for silver-based nanoparticles formation. Silver nanoparticles were obtained by a sustainable method by contact of silver nitrate and silica in the presence of ethanol. The successive UV irradiation gave rise to a nanoparticle size reduction and to an increase in the SPR typical of silver nanoparticles species, demonstrating the occurrence of the photoactivation process. The characterization of composites Cab-O-Sil–Ag and Cab-O-Sil–Ag-irr showed that these composites are covered by crystals of AgNO_3_ of about 2 µm, accompanied by the presence of silver particles with nanometric dimensions. After Cab-O-Sil-Ag UV irradiation, the obtained Cab-O-Sil-Ag-irr composite displays an overall decrease in the sizes of silver nanoparticles. The silver release from oleogels was gradual and slow, but the amount of silver released from oleogel-2 is higher than that from other oleogels. These composites showed an antimicrobial activity as good as AgNO_3_ and a lower cytotoxicity; thus, they were successively formulated in oleogels. The antimicrobial activity of the formulations was compared to that of the oleogels containing AgNO_3_ with the same silver concentration (oleogel-2 and oleogel-3).

The results were very interesting for oleogels containing silver–silica composites (oleogel-4 and oleogel-5). In fact, they showed good antimicrobial activity and resulted no or very low cytotoxic for fibroblasts and keratinocytes. The oleogel containing irradiated silver–silica composites (oleogel-5) showed reduced cytotoxicity, maybe due to stronger interactions between the matrix and silver and thus a lower silver dissolution. Oleogel-3 showed good antimicrobial activity and low cytotoxicity as well, but it resulted unstable and showed unacceptable organoleptic properties. As concerns oleogel-2, it showed good antimicrobial activity, but resulted cytotoxic maybe due to the higher silver ions’ release. In conclusion, the best performance was obtained for the oleogels containing silica–silver composites. The remarkable results are that these oleogels join the presence of both moisturizing agents, able to maintain skin hydration, and silver-based composites, able to exert antimicrobial activity while free of cytotoxic activity.

## Figures and Tables

**Table 1:** Oleogel composition expressed in percentage.

Excipient	Oleogel-1	Oleogel-2	Oleogel-3	Oleogel-4	Oleogel-5
Cab-O-Sil	5.000	5.000	4.883	4.880	4.880
Cab-O-Sil-Ag	-	-	-	0.120	-
Cab-O-Sil-Ag-Irr	-	-	-	-	0.120
AgNO_3_	-	0.016	0.016	-	-
Deionized water	-	-	0.100	-	-
Lanolin alcohols	-	-	0.500	-	-
Liquid paraffin	95.00	94.984	94.501	95.000	95.000

**Table 2:** MIC and MIC_silver_ values of the prepared composites.

Sample	*S. epidermidis*		*S. aureus*		*P. aeruginosa*	
	MIC (µg/mL)	MIC_silver_ (µg/mL)	MIC (µg/mL)	MIC_silver_ (µg/mL)	MIC (µg/mL)	MIC_silver_ (µg/mL)
Cab-O-Sil	>10,000	>10,000	>10,000	>10,000	>10,000	>10,000
Cab-O-Sil-Ag	38.7	3.2	77.5	6.5	155	13.2
Cab-O-Sil-Ag-irr	38.7	3.2	57.8	4.8	154	12.9
AgNO_3_	15.6	9.9	15.6	9.9	15.6	9.9
Gentamicin	0.1	0.1	<0.1	<0.1	<0.1	<0.1

**Table 3:** Cytotoxic activity of the composites against NCTC 2544 and HuDe. Data are the media of three independent experiments and are normalized for the silver content.

Sample	CC_50_ (µg/mL) NCTC 2544		CC_50_ (µg/mL) HuDe	
	4 h	24 h	4 h	24 h
Cab-O-Sil-Ag	17.48	22.06	13.43	16.35
Cab-O-Sil-Ag-irr	22.00	15.29	15.38	5.09
AgNO_3_	5.72	5.72	3.00	3.00

**Table 4:** Cytotoxicity of prepared oleogels on NCTC2544 and HuDe detected after 4 h and 24 h of contact of the oleogel with cell monolayers.

Sample	NCTC2544 4 h µg/mL	NCTC2544 24 h µg/mL	Hude 4 h µg/mL	Hude 24 h µg/mL
Oleogel-2	136	>250	75	>250
Oleogel-3	>250	>250	>250	>250
Oleogel-4	>250	>250	109	>250
Oleogel-5	>250	>250	>250	>250

## Data Availability

Data are contained within the article and Supplementary Materials.

## Supplementary Materials

The following supporting information can be downloaded at: https://www.mdpi.com/article/10.3390/jfb15010004/s1, Figure S1. XRD of Cab-O-Sil/AgNO_3_ physical mixture (a), Cab-O-Sil–Ag (b) and Cab-O-Sil–Ag-irr (c). The reflections are ascribable to the orthorhombic phase of AgNO_3_ (COD 1509468). Figure S2. UV-Vis absorbance spectrum of Cab-O-Sil solid powder in the Kubelka–Munk unit. Figure S3. ATR-IR spectra of Cab-O-Sil (black line), Cab-O-Sil-Ag (red line) and Cab-O-Sil-Ag-irr (blue line). Figure S4. TEM image (A, scale bar: 100 nm) and size distribution (B) of Cab-O-Sil. Figure S5. Just prepared oleogel images. Figure S6. Oleogel images after 3 month of storage at room temperature.
