# Supplementary material for: Antimicrobial Oleogel Containing Sustainably Prepared Silver-Based Nanomaterials for Topical Application
Source: J Funct Biomater. 2023 Dec 20;15(1):4. doi: 10.3390/jfb15010004 (PMC10817458; doi:10.3390/jfb15010004)
Supplement: Supplementary file 1 [file jfb-15-00004-s001.zip › jfb-2726554-supplementary.pdf]

Journal of
Functional Biomaterials

Article

Antimicrobial Oleogel Containing Sustainably Prepared Silver-Based Nanomaterials for Topical Application

Valeria Ambrogi [1,*], Morena Nocchetti [1], Donatella Pietrella [2], Giulia Quaglia [3], Alessandro Di Michele [4] and Loredana Latterini [3]

1 Dipartimento di Scienze Farmaceutiche, University of Perugia, Via del Liceo 1, 06123 Perugia, Italy; morena.nocchetti@unipg.it
2 Nano4Light Lab, Dipartimento di Chimica, Biologia e Biotecnologie, University of Perugia, Via Elce di Sotto 8, 06123 Perugia, Italy; donatella.pietrella@unipg.it
3 Dipartimento di Medicina e Chirurgia, University of Perugia, Piazzale Lucio Severi 1, 06129 Perugia, Italy; quagliagiulia09@gmail.com (G.Q.); loredana.latterini@unipg.it (L.L.)
4 Dipartimento di Fisica e Geologia, University of Perugia, Via Pascoli, 06123 Perugia, Italy; alessandro.dimichele@unipg.it
* Correspondence: valeria.ambrogi@unipg.it; Tel.: +39-075-5855125

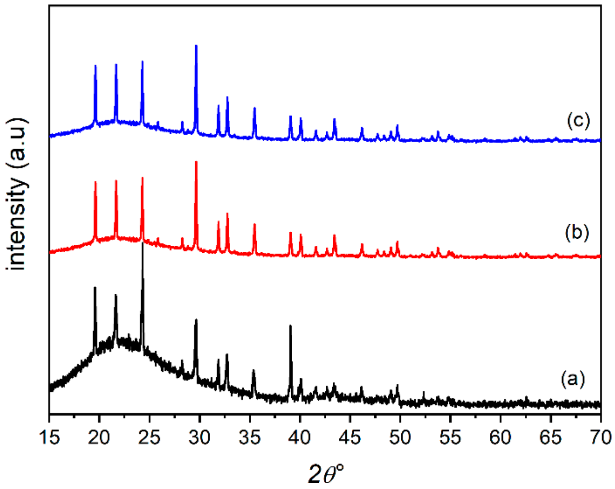

Figure S1. XRD of Cab-O-Sil/$AgNO_3$ physical mixture (a), Cab-O-Sil–Ag (b) and Cab-O-Sil–Ag-irr (c). The reflections are ascribable to orthorhombic phase of $AgNO_3$ (COD 1509468).

Citation: Ambrogi, V.; Nocchetti, M.; Pietrella, D.; Quaglia, G.; Di Michele, A.; Latterini, L. Antimicrobial Oleogel Containing Sustainably Prepared Silver-Based Nanomaterials for Topical Application. *J. Funct. Biomater.* **2023**, *15*, 4. https://doi.org/10.3390/jfb15010004

Academic Editors: Gopalu Karunakaran and Pankaj Vadgama

Received: 3 November 2023
Revised: 10 December 2023
Accepted: 15 December 2023
Published: 20 December 2023

Copyright: © 2023 by the authors. Licensee MDPI, Basel, Switzerland. This article is an open access article distributed under the terms and conditions of the Creative Commons Attribution (CC BY) license (https://creativecommons.org/licenses/by/4.0/).

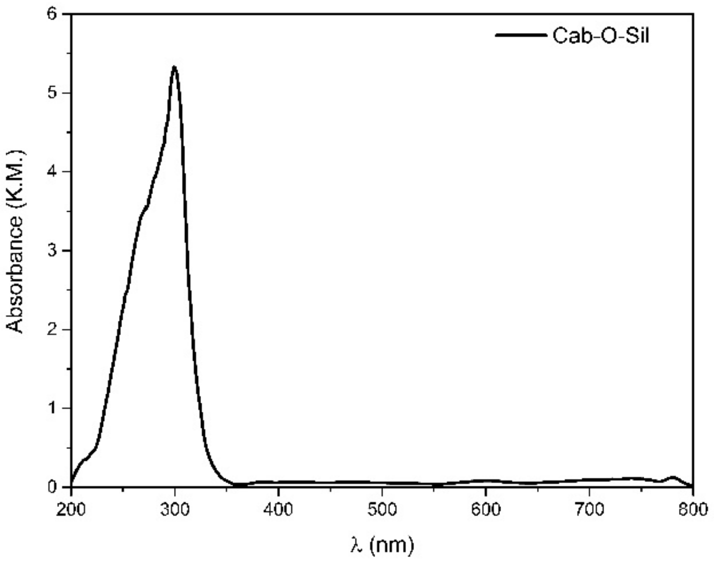

Figure S2. UV-Vis absorbance spectrum of Cab-O-Sil solid powder in Kubelka-Munk unit.

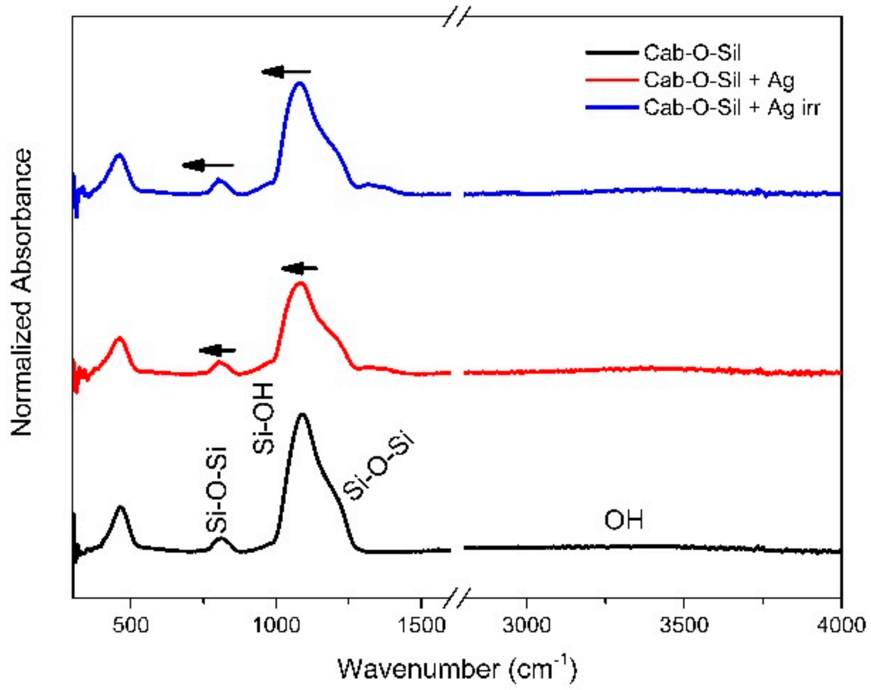

Figure S3. ATR-IR spectra of Cab-O-Sil (black line), Cab-O-Sil-Ag (red line) and Cab-O-Sil-Ag-irr (blue line).

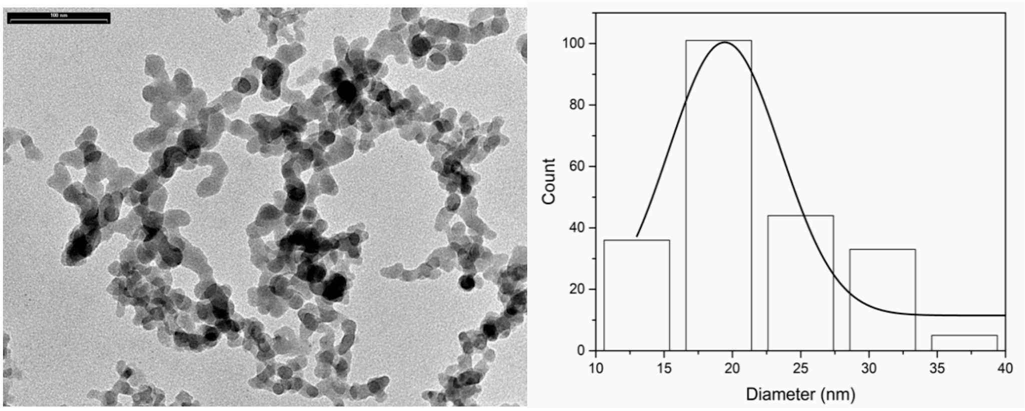

Figure S4. TEM image (A, scale bar: 100 nm) and size distribution (B) of Cab-O-Sil.

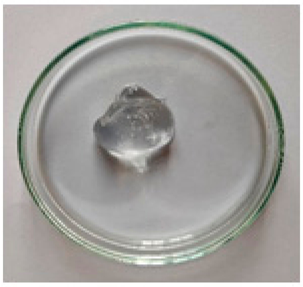

Oleogel-1

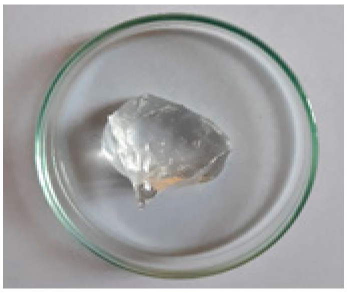

Oleogel-2

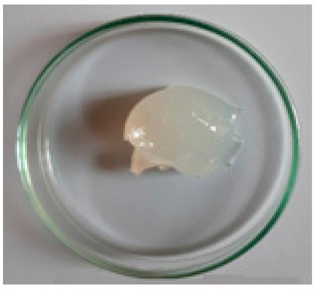

Oleogel-3

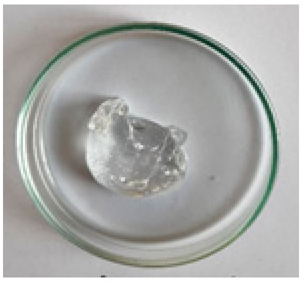

Oleogel-4

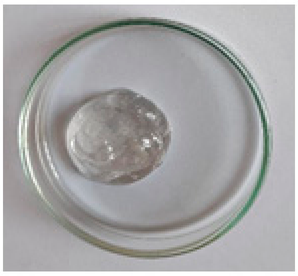

Oleogel-5

Figure S5. Just prepared oleogel images.

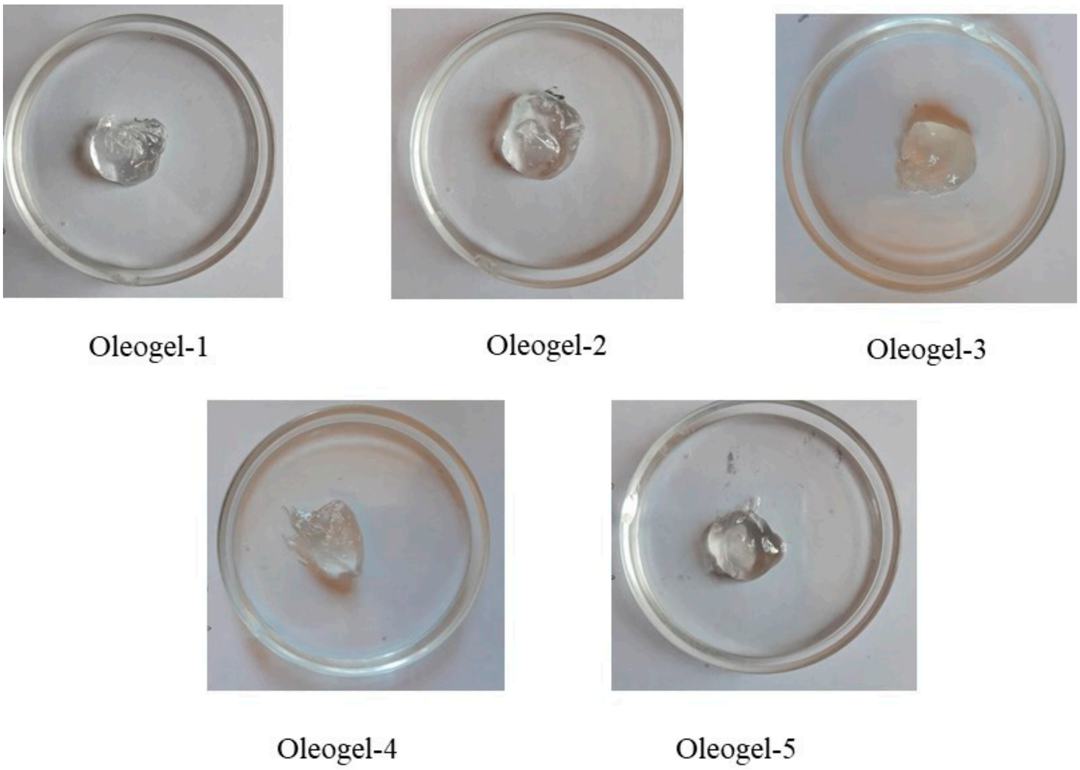

Figure S6. Oleogel images after 3 month of storage at room temperature.
